# Laboratory validation of the automated diagnosis of intestinal parasites via fecal sample processing for the recovery of intestinal parasites through the dissolved air flotation technique

**DOI:** 10.1186/s13071-024-06434-y

**Published:** 2024-08-30

**Authors:** Felipe Augusto Soares, Celso Tetsuo Nagase Suzuki, Edvaldo Sabadini, Alexandre Xavier Falcão, Amanda de Oliveira Baccin, Leyva Cecília Vieira de Melo, Jancarlo Ferreira Gomes

**Affiliations:** 1 School of Medical Sciences, Universidade Estadual de Campinas, Campinas, SP Brazil; 2 Laboratory of Image Data Science (LIDS), Institute of Computing (IC), Universidade Estadual de Campinas, Campinas, SP Brazil; 3 ImmunoCamp Science and Technology, Vinhedo, SP Brazil; 4 Department of PhysicalChemistry, Institute of Chemistry, Universidade Estadual de Campinas, Campinas, SP Brazil; 5 Adolfo Lutz Institute, São Paulo, Brazil

**Keywords:** Parasitic intestinal diseases, Dissolved air flotation, Diagnostic techniques and procedures, Artificial intelligence

## Abstract

**Background:**

Techniques for diagnosing intestinal parasites need technological advancements in the preanalytical (collection/processing) and analytical (detection) stages. The dissolved air flotation (DAF) technique effectively recovers parasites from processed feces for routine diagnosis. Artificial intelligence (AI) is a practical and affordable alternative to modernize the analysis stage of microscopy images and generates high efficiency in the parasitological examination of feces.

**Methods:**

The objective of this study was to standardize a laboratory protocol for stool processing using the DAF technique in conjunction with an automated diagnosis of intestinal parasites (DAPI) system. A total of 400 samples were obtained to perform the tests with the use of DAF to verify the recovery of the parasites as a function of the chemical reagent (polymer and surfactant), the volume of the flotation tube, and standardization of smear assembly on a microscopy slide, with automated analysis by DAPI. The DAF protocol that obtained the most satisfactory results in terms of parasite recovery (*P* < 0.05) and slide positivity was compared with the Three Fecal Test (TF-Test) protocol with manual (microscopists) and automated (DAPI) evaluation. We compared the sensitivity with the modified TF-Test technical protocol and the diagnostic agreement with the gold standard (Kappa) result.

**Results:**

There was no significant difference in the parasite recovery between the 10 ml and 50 ml tubes (*P* > 0.05). The surfactants showed a range of parasite recoveries between 41.9% and 91.2% in the float supernatant. We obtained a maximum positivity of 73% of the assembled slides when we applied DAF processing with 7% CTAB surfactant and 57% positivity with the modified TF-Test technique. Regarding diagnostic performance, the TF-Test-modified and DAF techniques used in fecal processing for subsequent computerized analysis by AI presented sensitivities of 86% and 94%, with kappa agreements of 0.62 and 0.80 (substantial), respectively.

**Conclusions:**

The DAF protocol defined in this study and the DAPI system are innovative processes for parasite recovery and fecal debris elimination that are favorable for effectively detecting pathogenic structures in laboratory diagnosis.

**Graphical Abstract:**

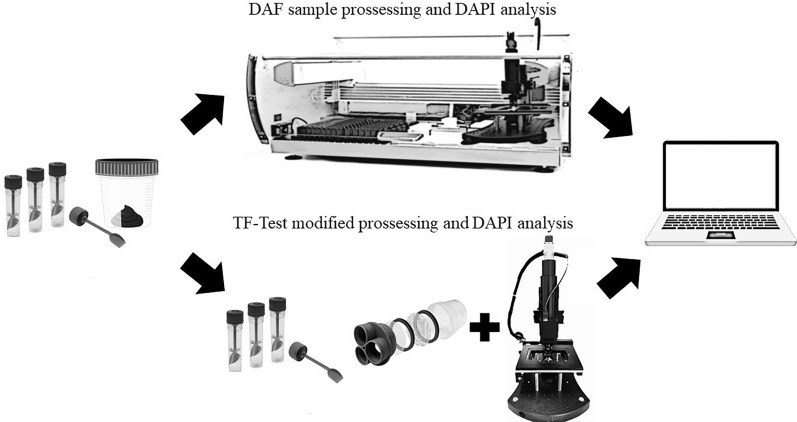

## Background

Intestinal parasitosis is a set of infectious diseases of the gastrointestinal system that affect millions of people, mainly in populations of social and economic vulnerability [1]. The diagnostic techniques used in laboratory routines for these diseases are based on conventional parasitological techniques with reduced efficiency for helminth and protozoa detection in infected hosts with low parasite loads [2]. New technical protocols for processing fecal samples and recovering parasites in feces on the basis of the physicochemical principles of separating solid and liquid compounds have been developed to overcome the current deficiencies [3–5].

The advancement of artificial intelligence (AI) in disease diagnosis methods has become essential for optimizing the results obtained by manual techniques, such as microscopic analyses that determine pathological conditions or bacterial, viral, or parasitic infections. Currently, new systems based on the capture of images by automated scanning of microscopy slides and the application of techniques such as artificial neural network (ANN), image foresting transform (IFT), and other methods for the identification and classification of structures have shown sensitivities between 74% and 99% for the simultaneous detection of up to 15 species of parasites [6, 7]. Mathison et al. [8] achieved a favorable agreement of 98% with the manual detection of intestinal protozoa using a convolutional neural network (CNN) model. However, these computational methods only achieve these diagnostic indices by applying protocols that process fecal samples to recover parasites and prepare thick droplets on microscopy slides with high elimination of fecal debris [9]. The modified Three Fecal Test (TF-Test) protocol was initially standardized as a fecal processing technique for the production of fecal smears to be analyzed by an automated image analysis system named automated diagnosis of intestinal parasites (DAPI) for the detection of parasites in samples [9, 10].

As in the environmental engineering and mining sectors, the dissolved air flotation (DAF) process has demonstrated applicability in processing stool samples to diagnose intestinal parasites. This new procedure obtained rates ranging from 48% to 92% in the recovery of the parasites and demonstrated a sensitivity of 91% in detecting these organisms via manual microscopic analysis [11].

Any device that reduces uncertainty about the state of the disease is considered a diagnostic test. There is no gold standard diagnostic method for detecting several intestinal parasites simultaneously in the same test. Thus, microscopy is still the recommended and low-cost method [12, 13]. New studies have been advancing with protocols and computational analysis systems for the diagnosis of domestic animal infections, such as Parasight All-in-One, VETSCAN IMAGYST, and DAPI, showing a sensitivity of 92%, 75–100%, and 81%, respectively [14–16]. Given the need for more advanced methods in clinical parasitology, the DAF-based stool processing protocol can be an alternative for integration with the DAPI system, thus reducing the chances of human error in stool processing and the extensive routine of microscopic analysis. Our objective in this study was to define a protocol for processing fecal samples with the DAF technique and to validate this protocol in the laboratory in conjunction with automated diagnosis by computational image analysis.

## Methods

### Sampling and ethical aspects

Stool samples were obtained from individuals who routinely attended the Clinical Analysis Laboratory of the Ouro Verde Hospital, located in Campinas, São Paulo, Brazil. Among the 400 samples analyzed, 300 stool samples positive for intestinal parasite infections were collected during the period of execution of the research, which took place at the Laboratory of Image Data Science (LIDS) of the Institute of Computing of the Universidade Estadual de Campinas (UNICAMP). All sample identifications were codified, patient anonymity was preserved, and no other personal information was used in this study.

All biological material was collected in duplicate on alternate days using three collection tubes from the TF-Test^®^ kit. One sample was used for the standard processing of the TF-Test technical protocol, and the other was used for the standardization tests of the DAF protocol with the DAPI system.

### Standardization of the protocol for sample processing and preparation of microscopy slides for automated diagnosis

The DAF device consists of an air saturation chamber (Jartest 218-3LDB, Ethik Technology, São Paulo, Brazil), an air compressor (BCP390/SCN, Biomec, Paraná, Brazil), and a rack for flotation tubes. The DAPI system has a computer, a motorized optical microscope with a digital camera, and software that interfaces to automatically control the microscope, capture images from the microscopy slides, and analyze the images obtained (Fig. 1).

**﻿Fig. 1:**
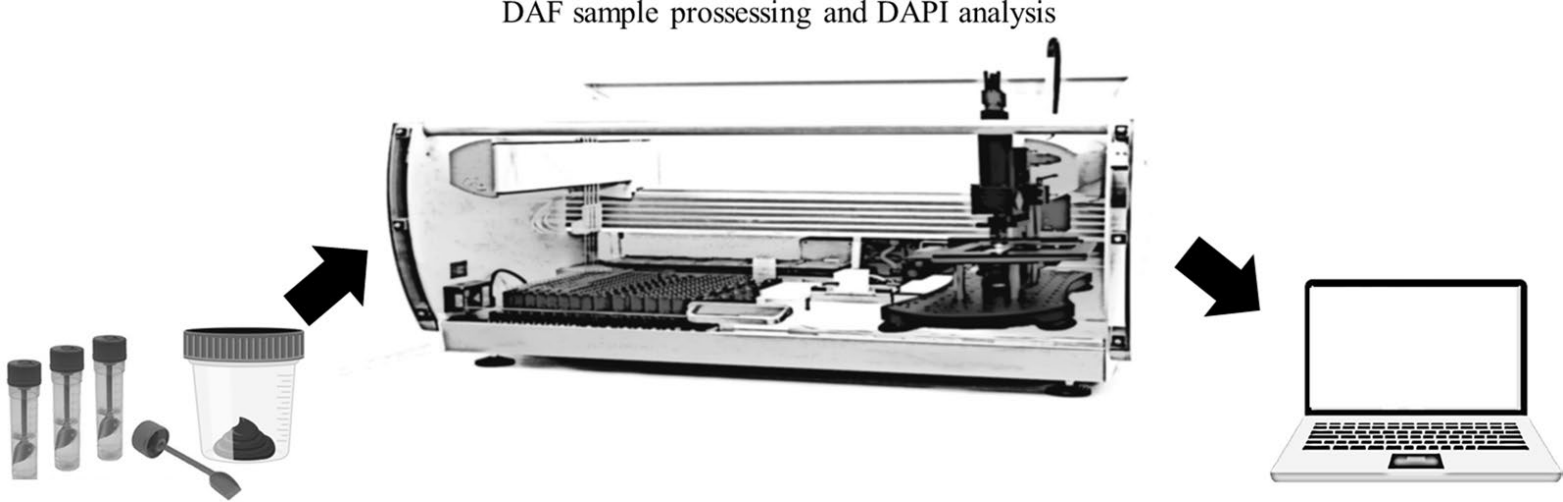
DAF device in conjunction with the DAPI system

The DAF technical protocol for the processing of stool samples used in this study involves the following steps: (a) the saturation chamber was filled with 500 ml of treated water containing 2.5 ml of the surfactant hexadecyltrimethylammonium bromide and was pressurized under a pressure of 5 bar with a saturation time of 15 min; (b) the biological material was collected in a 300 mg portion in each of the three collection tubes of the TF-Test parasitological kit on alternate days to evaluate a total amount of approximately 900 mg of fecal sample, according to the manufacturer’s instructions; (c) the collection tubes were coupled to a set of filters containing a filter mesh with orifices of 400 μm and 200 μm in diameter, and this set was agitated for 10 s in vortex equipment, promoting the mechanical filtration of the fecal contents; (d) the 9 ml filtered sample volume was transferred to a test tube (10 ml and 50 ml); (e) the depressurization system was inserted utilizing a cannula device in the lower part of the tubes, and saturated fractions of 1 ml and 5 ml (10%) were injected into these tubes, the rack can support up to 20 tubes for simultaneous processing; (f) after 3 min of microbubble action, 0.5 ml of the floated sample was retrieved from the supernatant region of the tube using a Pasteur pipette and transferred to a microcentrifuge tube containing 0.5 ml of ethyl alcohol; and (g) to prepare the fecal smear, the recovered sample was homogenized with a Pasteur pipette, a 20 μL aliquot was transferred to a microscope slide, and soon after, the smear received a volume of 40 μl of 15% Lugol’s dye solution and 40 μl of saline solution (or distilled water) for observation under a conventional light optical microscope.

To define a standardized protocol for computational analysis, we performed tests with the volume of the flotation tube, type/concentration of surfactant or polymer, and preparation of the microscopy slides to obtain maximum recovery of parasites and consequently reflect a greater probability of positivity in the slides (Fig. 2). The laboratory protocol for processing stool samples and subsequent diagnosis using conventional and manual light microscopy was previously validated by Soares et al. [11] and used as the standard for the following tests.

**﻿Fig. 2:**
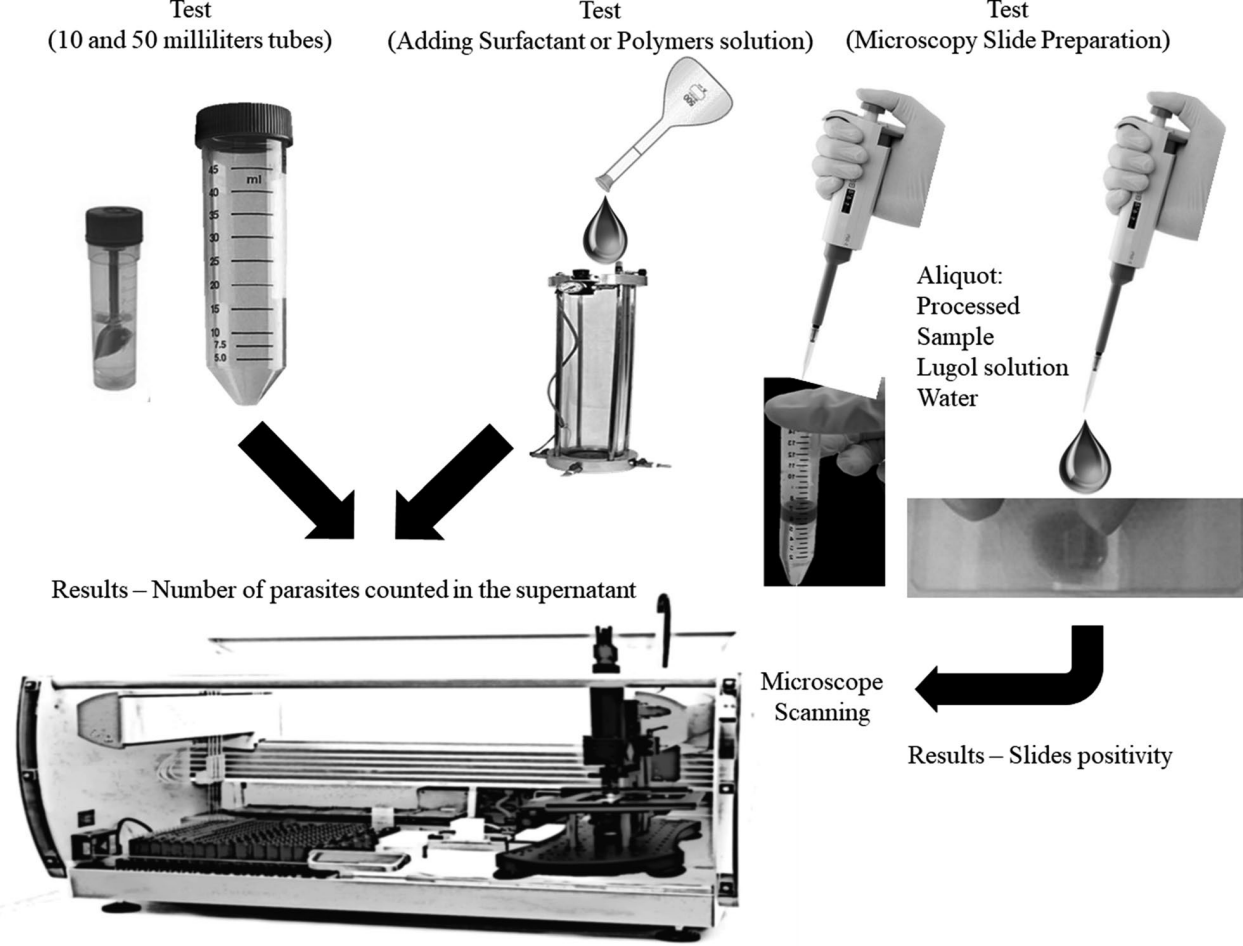
Study design of the standardization stages of the DAF protocol

#### Parasite recovery test in 10 ml and 50 ml tubes

To define the type of flotation tube suitable for the recovery of parasites in the contents floated with the microbubbles in the supernatant region of the tube, we evaluated 10 ml test tubes and 50 ml Falcon tubes in the DAF protocol, and nine stool samples (three for each species) containing the species *Ascaris lumbricoides*, *Strongyloides stercoralis*, and *Giardia duodenalis* were homogenized and separated in duplicate in the collection tubes of the TF-Test kit to be processed by the DAF protocol. Each sample was divided into three tests (triplicate). After the microbubbles were inserted into the 10 ml and 50 ml tubes evaluated, the floated content (2 ml) was recovered from the supernatant region of the tubes, and the tube was subsequently centrifuged to obtain the nonfloated content in the sediment of each tube evaluated. The floated and nonfloated fecal material of the tubes (10 ml and 50 ml) was analyzed under a microscope, and the eggs and larvae (*A. lumbricoides* and *S. stercoralis*) of the parasites were counted manually to obtain the percentage of parasite recovery in the tube supernatant. The cysts of *G. duodenalis* were quantified in five central fields of the slide to get a total of cysts counted in the floated (supernatant) and nonfloated (sediment) samples. Thus, we defined the approximate percentage of recovery of the cysts in the tubes evaluated.

#### Testing of surface load-modifying polymers and surfactants for parasite recovery

To evaluate the efficiency of parasite recovery with the use of charge-modifying chemical reagents, the cationic surfactants hexadecyltrimethylammonium bromide (CTAB) and cetylpyridinium chloride (CPC) were used, both at a concentration of 10%, and the cationic polymers poly dialyl dimethylammonium chloride (PolyDADMAC—molecular weight < 100,000) were used at a concentration of 0.25%, chitosan at a concentration of 1%, and hydroxyeltyl cellulose (Natrosol Plus 330) at a concentration of 1%. Each reagent was evaluated separately by adding 2.5 ml of surfactant or polymer to 500 ml of distilled water inside the saturation chamber. The liquid has been saturated with air and has produced positively charged microbubbles at its gas–liquid interface. The same method of quantification of eggs, larvae, and cysts as in the previous test was applied to estimate the percentage of recovery of parasitic structures using chemical reagents. In this test, 12 samples (3 for each species) containing *A. lumbricoides*, *S. stercoralis*, *G. duodenalis*, and *Schistosoma mansoni* were homogenized and placed in tubes for each reagent test.

#### Microscopy slide preparation test

The surfactants CTAB and CPC were used as the main reagents to obtain parasites via flotation. For this test, 10% and 7% CTAB and 5% CPC were used to verify the positivity of the slides when they were automatically analyzed. The DAPI system was used to perform automated complete slide scanning, and 2000 images/slides were analyzed in 3 min. For this purpose, the fecal sample obtained from the flotation of the reagent assays was transferred in a volume of 300 μl to empty microcentrifuge tubes and a microcentrifuge tube containing 300 μL of 70% ethyl alcohol and homogenized for 10 s in a vortex. The microscopy slides were prepared with fecal smears with proportions of 20 μl of fecal sample, 40 μl of 15% Lugol’s solution, and 40 μl of treated water. All slides prepared for analysis after DAF processing (test) were compared for positivity (positive–negative) about slides prepared using the modified TF-Test technical protocol, used as a standard in DAPI. In this test, 42 positive samples were mixed in 10 tubes to be processed, and 30 slides were prepared for each protocol test.

### Intralaboratory validation of the technical protocol of microscopic processing and reading for the diagnosis of intestinal parasites

For laboratory (intralaboratory) validation, samples screened by direct examination were used to simultaneously apply the modified TF-Test technique and the technical protocol with DAF after standardizing the optimal parameters obtained in the testing phase. A set of 73 fecal samples positive and negative for intestinal parasite infections were used for a comparative evaluation between the fecal processing protocols with the modified TF-Test [17] and the dissolved air flotation as the preanalytical step, and the microscopy slides with the fecal smear resulting from both protocols were analyzed by the DAPI automated diagnostic system as the analytical step of this test. The slides prepared with fecal smears obtained by the modified TF-Test protocol were manually analyzed by two experts at a defined maximum time of 3 min/slide, and the results obtained manually were compared with the results recorded in the DAPI system and audited by two experts. This study recorded the concordance of the true positive (TP) and true negative (TN) data obtained in the two processes as the gold standard result.

### Statistical analysis

The mean percentages of parasites recovered in the floated region in the control assays were compared with those in the assays utilizing the bilateral parametric *t*-test from Student’s *t*-test and analysis of variance (ANOVA) with a 95% confidence interval. These results were compared to evaluate the sensitivity, specificity, and *kappa* (*k*) coefficient and classified as almost perfect, substantial, moderate, weak, or poor [18, 19]. The software used for the statistical evaluations was *BioEstat* 5.3, version *Windows©* 8, for the Comparative Evaluation of Technical Protocols *TF-Test* and DAF.

## Results

Of the 400 samples obtained, 300 were positive (66.6%) for some intestinal parasites, and 136 were used in laboratory tests to validate automated processing and diagnosis.

In tests with 10 ml and 50 ml flotation tubes for parasite recovery, a slightly greater recovery was observed using 50 mL tubes, although not statistically significant (Table 1). Although the parasites’ recovery did not significantly differ for either tube (10 ml or 50 ml), the percentage differences may reduce the chances of positive slides being prepared for microscopic analysis.

**Table 1:** Average percentage of parasite recovery as a function of the flotation tube (10 ml and 50 ml) (*n* = 9)

Species	Tube 50 ml	Tube 10 ml
*A. lumbridoides*	74.30	66.63
*S. stercoralis*	81.34	72.05
*G. duodenalis*	30.29	35.00
Average	62	58
*P-value*	0.45	

When evaluating the recovery performance of parasitic structures (eggs, larvae, and cysts) in triplicate, we noticed that helminth eggs and larvae were rescued in the floated supernatant by more than 50% on average, except for the polymers chitosan and natrosol. *G. duodenalis* cysts were more concentrated in the supernatant using CTAB and CPC surfactants, with 48.44% and 41.96% recovery, respectively (Table 2).

**Table 2:** Average percentage of parasitic structures floated in relation to the surfactant or microbubble charge modifying polymer in the DAF assays in triplicates (*n* = 12)

Experiment		*A. lumbricoides*	*S. mansoni*	*G. duodenalis*	*S. stercoralis*
		*% floated*	*% floated*	*% floated*	*% floated*
CTAB	1	93.17	72.34	46.94	91.63
	2	82.71	64.38	51.44	85.75
	3	88.32	61.54	46.94	96.39
	Average	88.07	66.09	48.44	91.26
CPC	1	76.38	–	41.83	69.23
	2	92.58	69.57	43.00	65.38
	3	89.69	76.47	41.05	72.00
	Average	86.21	73.02	41.96	68.87
Quitosana	1	57.54	51.22	18.31	88.60
	2	42.75	30.61	36.66	76.32
	3	52.33	60.42	43.59	72.36
	Average	50.87*	47.42*	32.86	79.09
PDADMAC	1	77.59	76.00	–	86.67
	2	75.57	73.81	–	74.42
	3	56.69	70.73	–	72.00
	Average	69.95	73.51	–	77.70
Natrosol	1	25.06	41.67	18.17	55.35
	2	30.96	50.88	29.22	36.96
	3	25.70	29.55	24.63	42.78
	Average	27.24*	40.70*	24.01*	45.03*

*P < 0.01

The evaluation of the positivity of the prepared slides employed the TF-Test modified (standard) and DAF processing protocols with the dilution of the sample in ethyl alcohol and without this dilution. Ethyl alcohol aids in dispersing surfactant residue, preventing aggregation of biological components in the sample.

In the first tests, where we verified the positivity of the slides with positive samples for protozoa and intestinal helminths, we detected a more significant percentage of parasites in the DAF protocol with samples diluted in ethyl alcohol (56%) than in the modified TF-Test protocol (53%) (Table 3). Overall, protozoa were detected in 60% of the patients, whereas helminths were detected in 39%.

**Table 3:** Positivity of slides prepared by the TF-Test modified and DAF technical protocols analyzed using the DAPI system (*n* = 22 positive)

Sample	TF-Test modified		DAF (CTAB 10%)				DAF (CTAB 10%)		
	20 µl sediment		20 µl sediment				20 µl sediment + alcohol ethyl (1:1)		
	Slide		Slide				Slide		
	1	2	3	1	2	3	1	2	3
1	EC/G	EC/G	EC/G	EC/G	EC/G	EC/G	EC/G	EC/G	EC/G
2	EN/TA	EN	EN	EN	EN	EN	EN/G	EN	EN/TA
3	0	0	EV	0	EV	0	0	EV	0
4	0	0	0	0	0	HN	HN	0	0
5	AL	0	AL/ST/G	AL/ST	AL/ST	AL/ST/EC	AL/ST	AL/ST	AL/ST/EC
6	EC/EN	EC/EV	0	0	0	EN/EC/G	EN/EC/G	EN/EC/G	EN/EC/G
7	G	G	G	0	0	G	G	G	G
8	EV	0	EV	0	0	0	0	0	0
9	EH	EH	EH	0	EH	EH	EH	EH	0
10	EN/G/EC/AL	0	EN/G/EC/AL	G/EC/EN	G	G/EC/EN	0	0	EC/G/AL
Positive slides	66								
Positivity	35		31				38		
Positivity (%) protozooa	60		55				64		
Positivity (%) helminths	42		33				42		
Total percentage	53		47				56		

AL, *Ascaris lumbricoides*; EC, *Entamoeba coli*; EH, *Entamoeba histolytica/dispar/moshkovskii complex*; EN, *Endolimax nana*; EV, *Enterobius vermicularis*; G *Giardia duodenalis*; HN *Hymenolepis nana*; TA, Taenia spp.; ST *Strongyloides stercoralis*

In the slide positivity tests with the application of the modified TF-Test and DAF protocols (CTAB 7%, CPC 5%), we observed a higher slide positivity (73%) with the DAF protocol with the surfactant CTAB 7% and dilution of the sample in ethyl alcohol (Table 4).

**Table 4:** Positivity of slides prepared using the TF-Test modified and DAF technical protocols with CTAB and CPC surfactants analyzed using the DAPI system (*n* = 20 positive)

Sample	TF-Test modified			DAF (CTAB 7%)			DAF (CPC 5%)		
	20 µl sediment			20 µl sediment + alcohol ethyl (1:1)			20 µl sediment + alcohol ethyl (1:1)		
	Slide			Slide			Slide		
	1	2	3	1	2	3	1	2	3
1	EC/IO	EC/IO	EC/IO	EC/IO	EC/IO	EC/IO	EC	IO	EC/IO
2	0	0	0	0	0	0	0	0	0
3	0	EN	EN	EN	EN	EN	EN	0	EN
4	EV/EC	EC/EN	EC/EN	EC/G/EN	EC/G/EN	EC/G/EN	EC/G/EN	EC	EC/EN
5	0	0	EV	0	0	EV	EV	0	EV
6	AL/G/EC	G	AL/G/EC	G	AL/G	AL/G/EC	G	G/EC	G
7	0	0	0	0	0	0	0	0	0
8	G/EC	EC/G/EN	EC	EV/EC/G	EV/EC/G	EV/EC/G	AL/EC	0	EC/G
9	AL/TT	EN/AL	EN/AL	AL/HN/EN	AL/EN	AL/HN/EN	AL/HN/EN	AL	AL/HN/EN
10	0	0	0	0	ANC	ANC	0	0	0
Positive slides	60								
Positive slides	34			44			29		
Positivity (%)	57			73			48		

AL, *Ascaris lumbricoides*; ANC, Ancilostomídeo/Hookworm; EC, *Entamoeba coli*; EN, *Endolimax nana*; EV, *Enterobius vermicularis*; G, *Giardia duodenalis*; HN, *Hymenolepis nana*; IO, *Iodamoeba bütschlii*; TT *Trichuris trichiura*

**Fig. 3:**
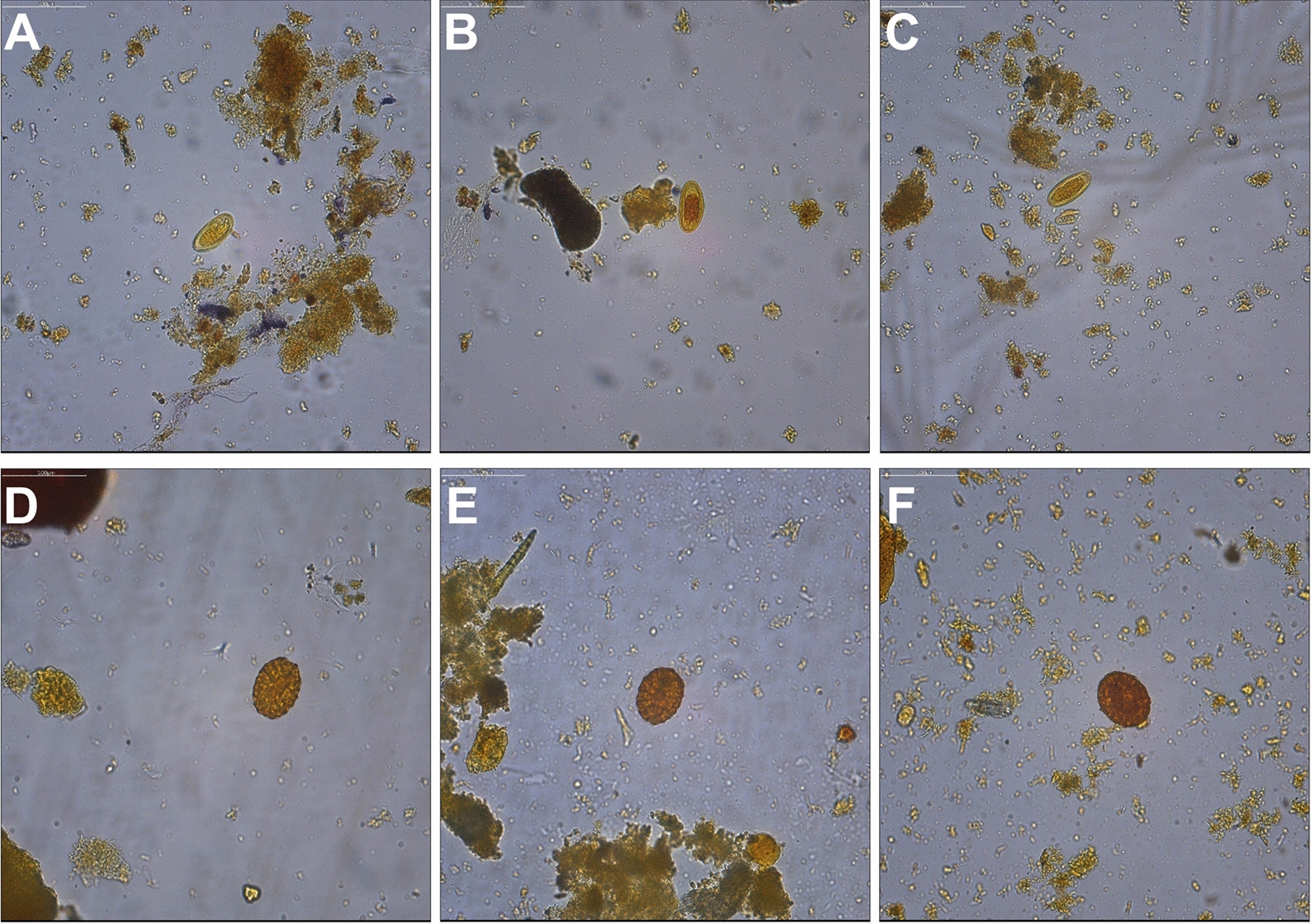
Images of *E. vermicularis* eggs (top row) and *A. lumbricoides eggs* (bottom row) obtained using the modified fecal processing protocols of the **A**–**D** TF-Test: **B**–**E** DAF with 5% CPC surfactant and **C**–**F** DAF with 7% CTAB surfactant

The DAF protocols using the 5% CPC surfactant and modified TF-Test were less sensitive for detecting hookworm eggs than the DAF protocol with the 7% CTAB surfactant. The performance of the processing protocol with DAF was satisfactory in rescuing the parasites in terms of their total morphological integrity and with little interference from organic debris in the images obtained by the automated system (Fig. 3), similar to the images generated with the modified TF-Test protocol.

In the intralaboratory validation of the fecal sample processing (FAD) protocol with automated analysis (DAPI) compared with the modified TF-Test protocol in manual (microscopists) and automated analysis, we noticed that processing using DAF obtained the highest sensitivity (94%), accuracy (95%), and kappa (0.80—substantial) indices in relation to the other sample analysis protocols (Table 5).

**Table 5:** Comparative performance evaluation of fecal sample processing protocols and manual and automated diagnosis of intestinal parasites (*n* = 73)

Results	TF-Test MANUAL	TF-Test DAPI	DAF DAPI	Gold standard*
True positive	51	54	59	63
True negative	10	10	10	10
False negative	12	9	4	0
False positive	0	0	0	0
Sensitivity	81	86	94	
Specificity	100	100	100	
Accuracy	84%	88%	95%	
*Kappa*	0.54	0.62	0.80	
Interpretation	Moderate	Substantial	Substantial	

*Gold standard = TF-test and DAF-DAPI combined results

The standardized DAF-DAPI protocol in this study has the following steps:

(a) The saturation chamber was filled with 500 ml of treated water containing 2.5 ml of 7% CTAB surfactant and pressurized under a pressure of 5 bar with a saturation time of 15 min; (b) the biological material was collected in a 300 mg portion in each of the three collection tubes of the TF-Test parasitological kit on alternate days to evaluate a total amount of approximately 900 mg of fecal sample diluted in 9 ml of 7% buffered formalin; (c) the collection tubes were coupled to a filter set containing a filter mesh with holes of 400 μm and 200 μm in diameter, this set was agitated for 10 s in vortex equipment, promoting the fecal contents’ mechanical filtration; (d) a volume of 9 ml of the filtered sample was transferred to a flotation tube (Falcon© 50 ml) and filled with distilled water to a volume of 45 ml (water + fecal solution); (e) the depressurization system was inserted employing a cannula device in the lower part of the flotation tube, and a saturated fraction of 5 ml (10%) was injected into this tube; (f) after 3–5 min of microbubble action, 0.3 ml of the floated sample was removed from the supernatant region of the tube using a Pasteur pipette and transferred to a microcentrifuge tube containing 0.3 ml of ethyl alcohol; and (g) to prepare the fecal smear, the recovered sample was homogenized (agitated) in a tube, and an aliquot of 20 μl was transferred to a microscope slide. Soon after, the smear received a volume of 40 μl of Lugol’s dye solution and 40 μl of saline solution, and the slide was directed for analysis by the DAPI system.

## Discussion

In this study, we performed the first validation of a parasitological protocol for the processing of feces with the application of DAF in conjunction with automated computational analysis (DAPI) of microscopy slides, unlike all the technical procedures practiced in the examination of Ova and Parasites (O&P). Recent studies have sought to increase AI with image analysis through computational techniques involving segmentation and classification of objects for more accurate detection of parasites via routine examinations [6, 9, 20].

In the parasite recovery tests as a function of the volume of the flotation column used, there was no significant difference (*P* > 0.05) in the percentage of parasites obtained in the floated content (supernatant) of the 10 ml tube compared with the values obtained with the 50 ml tube. Considering that the samples in routine examinations can vary from low to high intensity of infection, this mean difference in recovery (Table 1) of 4% between the tubes may be a factor in reducing the sensitivity of the technique when there is low intensity of infection of the analyzed sample [21]. The tubes we use are composed of polypropylene; however, Rao et al. [22] demonstrated that the use of polypropylene flasks as the medium where flotation occurs should be avoided due to their hydrophobic surface characteristic, which is attractive to microbubbles to prevent their adhesion to flask walls and can reduce flotation efficiency. Rao et al. [22] suggested that flotation tubes with optimal height/diameter ratios in microalgae recovery (*Chlorella vulgaris*) should have proportions between 1.6 and 2.05, unlike the tubes we used, which have a ratio of 3.83. Thus, our results can be improved using tubes of nonhydrophobic material and more appropriate dimensions for the dwell time and interaction between particles and microbubbles.

In-depth knowledge about the physicochemical characteristics of intestinal parasite resistance structures is essential for identifying more effective chemical reagents for the techniques used in laboratory diagnosis. According to previous studies, the cysts of *G. duodenalis* and oocysts of *Cryptosporidium* spp. were characterized by a negative surface charge. The *Ascaris suum* eggs are hydrophobic and have a negative charge on their outer membranes [23–25]. Therefore, we evaluated the ability of filler-modifying polymers and surfactants (cationic) to reduce the repulsive loads between microbubble parasites and to increase the efficiency of capturing these structures by CTAB and CPC surfactants (Table 2). Henderson, Parsons, and Jefferson [26] obtained 74–96% microalgae recovery efficiency (*Microcystis aeruginosa*) using PolyDADMAC, similar to the results obtained with this low-molecular-weight polymer. We found that the average recovery of the PolyDADMAC polymer from the parasites was more significant than 70%. However, there was an increase in the adsorption of the parasites to the fecal debris, which prevented the counting of cysts of *G. duodenalis* and made it impossible to see them on the blade. The Natrosol polymer did not reach the minimum recovery of 50%, and chitosan did not get this minimum percentage in the two species analyzed, so we consider these reagents not applicable in this experimental situation.

The automated diagnostic system evaluated the slides prepared with samples processed using the DAF protocol predefined in this study. Due to the amount of debris and its adsorption to the structures of the parasites with surfactants at a concentration of 10%, we reduced the concentration of the CTAB surfactants to 7% and the CPC to 5% for the positivity test after mounting the slides. Recently, Boonyong et al. [6] evaluated the efficiency of AI in O&P examination and observed a low sensitivity of the FA280 system protocol when comparing the centrifugal sedimentation principle with that of ethyl acetate. This resulted in the detection of the parasites being 6% greater than the spontaneous sedimentation process applied by the FA280 system with automated analysis. In addition to Boonyong et al. [6], we noticed that slide positivity tends to decrease in triplicate slides due to factors such as a decrease in the intensity of eggs, larvae, or cysts in the final processed sample; variation in parasite coloration; variation in the initial focus plane captured automatically; and excess fecal debris that may overlap the parasites of diagnostic interest.

The test that obtained the best performance of slide positivity (73%) was the DAF stool processing protocol with the use of 7% CTAB, surpassing the modified TF-Test protocol (Table 4) previously standardized in the studies by Suzuki et al. [10] and Osaku et al. [9]. The images in Fig. 3 demonstrate the visual field similarity of the slides prepared using the TF-Test modified and DAF processing protocols with the surfactants automatically evaluated by DAPI. The elimination of debris in the fecal smear has repercussions on the evidence of parasitic structures without morphological alteration of the parasites, which is essential for increasing the sensitivity and accuracy of detection by AI, as demonstrated by the DAF procedure. Zeleke et al. [27] and Nikolay, Brooker, and Pullan [21] showed sensitivities of 44.3% and 53%, respectively, with the formaldehyde-ether concentration (FEC) procedure by centrifugation-sedimentation for the detection of hookworm eggs, which demonstrates that other principles of egg retrieval, such as DAF, are more appropriate for this detection.

The DAPI system presented in this study favors large-scale laboratory tests by maintaining consistency in performance. It performs the complete analysis of the slide/coverslip (2000 fields) in approximately 3 min, reduces human effort, allows a specialist confirmation of the result, and makes it possible to train the classification algorithm with the complement of the database (images), further improving the diagnostic performance. In this way, this system was standardized with a broad-spectrum fecal processing technique, unlike technical protocols such as the Baermann and Harada Mori procedures, which only cover the detection of nematode larvae present in feces, thus restricting the objective of broad detection of species, especially for routine laboratory use.

## Conclusions

These results confirm the application of DAF as an innovative process for parasite recovery and fecal debris elimination that is favorable for detecting pathogenic structures in laboratory diagnosis. The DAF protocol used in this study can be improved through structural changes to the DAF device that allow for more effective recovery of parasites in fecal samples and advance more sensitive computational algorithms to detect parasites.

## Data Availability

The data generated or analyzed during this study are included in the published article.
